# The anti‐dipsogenic and anti‐natriorexigenic effects of estradiol, but not the anti‐pressor effect, are lost in aged female rats

**DOI:** 10.14814/phy2.14948

**Published:** 2021-07-20

**Authors:** Jessica Santollo, Jason A. Collett, Andrea A. Edwards

**Affiliations:** ^1^ Department of Biology University of Kentucky Lexington KY USA; ^2^ Department of Anatomy, Cell Biology and Physiology Indiana University School of Medicine Indianapolis IN USA

**Keywords:** drinking, estrogen receptor, NaCl intake, water intake

## Abstract

Estradiol (E2) inhibits fluid intake in several species, which may help to defend fluid homeostasis by preventing excessive extracellular fluid volume. Although this phenomenon is well established using the rat model, it has only been studied directly in young adults. Because aging influences the neuronal sensitivity to E2 and the fluid intake effects of E2 are mediated in the brain, we tested the hypothesis that aging influences the fluid intake effects of E2 in female rats. To do so, we examined water and NaCl intake in addition to the pressor effect after central angiotensin II treatment in young (3–4 months), middle‐aged (10–12 months), and old (16–18 months) ovariectomized rats treated with estradiol benzoate (EB). As expected, EB treatment reduced water and NaCl intake in young rats. EB treatment, however, did not reduce water intake in old rats, nor did it reduce NaCl intake in middle‐aged or old rats. The ability of EB to reduce blood pressure was, in contrast, observed in all three age groups. Next, we also measured the gene expression of estrogen receptors (ERs) and the angiotensin type 1 receptor (AT1R) in the areas of the brain that control fluid balance. ERβ, G protein estrogen receptor (GPER), and AT1R were reduced in the paraventricular nucleus of the hypothalamus in middle‐aged and old rats, compared to young rats. These results suggest the estrogenic control of fluid intake is modified by age. Older animals lost the fluid intake effects of E2, which correlated with decreased ER and AT1R expression in the hypothalamus.

## Graphical abstract

The results of this study suggest that the estrogenic control of fluid intake is modified by age in female rats. Older animals lost the fluid intake effects of estradiol, which correlated with decreased estrogen receptor and angiotensin type 1 receptor expression in the paraventricular nucleus of the hypothalamus.

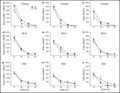

## 1 INTRODUCTION

It is well established that estradiol (E2) interacts with the renin–angiotensin system to regulate fluid homeostasis in females. E2 modulates both the fluid intake effects of angiotensin II (AngII) and the pressor effect (Curtis, 2009; Santollo & Daniels, 2015b), both of which are critical for maintaining and defending body fluid homeostasis. For instance, across the estrous cycle, AngII‐stimulated water and saline intakes are lowest on the day of estrus (Danielsen & Buggy, 1980; Findlay et al., 1979). This effect is mediated by E2, as hormone replacement studies demonstrate that E2 reduces AngII‐stimulated intake in ovariectomized (OVX) rats (Fregly & Thrasher, 1978; Jonklaas & Buggy, 1984; Santollo & Daniels, 2015a; Santollo et al., 2016), which is not modulated by progesterone (Kisley et al., 1999; Spiteri et al., 1980; Thrasher & Fregly, 1977). The pressor effect of AngII is mediated both peripherally by vasoconstriction and sympathetic activation and centrally through sympathetic activation and vasopressin release. E2 action dampens both the peripheral and central pressor effects of AngII (Jonklaas & Buggy, 1984; Xue et al., 2007, 2014). Together these inhibitory effects of E2 act to prevent excessive fluid volume, reduce blood pressure, and from a translational standpoint likely contribute to lower rates of hypertension in premenopausal women (Reckelhoff, 2001).

Aging influences neuronal sensitivity to E2‐positive feedback in females, particularly within the hypothalamus. For example, gonadotropin‐releasing hormone activation and the subsequent luteinizing hormone surge are attenuated and delayed in middle‐aged, compared to young adult, rats after E2 exposure (Brann & Mahesh, 2005; Buyuk et al., 2010; Kermath & Gore, 2012). In addition, genes in the hypothalamus, such as melanin‐concentrating hormone and proopiomelanocortin, lose sensitivity to E2 in middle‐aged female rats (Santollo et al., 2012). Whether aging influences the effects of E2 on fluid homeostasis, in contrast, has received little attention. The ability of ovarian hormones to reduce the AngII‐mediated pressor response has been reported to be lost in aged female (Go et al., 2013) mice; however, E2 was not directly manipulated in this study (Mirabito et al., 2014). Furthermore, to our knowledge, no studies have directly examined the effect of age on E2's regulation of the centrally mediated drinking and pressor effects of AngII.

It is critical to understand how the regulation of fluid homeostasis by E2 is altered as a function of age given the importance of proper fluid balance for cardiovascular function and that postmenopausal women are at an increased risk for cardiovascular‐related diseases (Go et al., 2013; Suzuki & Kondo, 2012; Writing Group M et al., 2016). We, therefore, investigated if aging influences E2's regulation of fluid homeostasis by measuring two key effects of AngII, fluid intake and the pressor response. We focused on central AngII administration to isolate the effect of the central pressor response and because AngII action in the brain stimulates fluid intake. To achieve these goals, we used young, middle‐aged, and old adult female rats to test the hypothesis that aging alters the anti‐dipsogenic, anti‐natriorexigenic, and anti‐pressor effect of E2 after central AngII‐treatment.

## 2 MATERIALS AND METHODS

### 2.1 Animals and housing

Female Sprague Dawley rats (Envigo Laboratories) were used in these experiments. Young (*n* = 12; 3–4 months) and middle‐aged (*n* = 12; retired breeders, 10–12 months) rats underwent surgery (see below) after 8–14 days of acclimation to the facility. Rats in the old group (*n* = 11; retired breeders, 18–19 months) arrived at the facility at 12 months old and aged for an additional 6 months before being used in these experiments. The use of retired breeders for the aged groups is consistent with previous studies examining the neuroendocrine controls of reproductive aging (Lederman et al., 2010; Neal‐Perry et al., 2008, 2009, 2014; Rubin et al., 1994). Rats were singly housed (after surgery) in modified shoebox cages which had two external lick blocks to allow for the connection of the bottle spouts with our custom contact lickometer (UKY Electronic Shop, Lexington, KY). Rats had *ad libitum* access to food (Teklad 2018; Harlan Laboratories), tap water, and 1.5% NaCl (0.26 M NaCl) solution unless otherwise noted. Body weight was monitored daily and vaginal cytology samples were collected, as previously described (Santollo et al., 2017), prior to ovariectomy surgery. All young and middle‐aged rats showed a 4–5 day estrous cycle, while all aged rats displayed repetitive pseudopregnant cycles (predominately leukocytes with cornified cells appearing once or twice every 14 days (Harman et al., 1985; Wise et al., 1991). On test days, young rats weighed between 221 and 286g, middle‐aged rats weighed between 268 and 338g, and old rats weighed between 260 and 370g. The temperature‐ and humidity‐controlled colony room was maintained on a 12:12 h light–dark cycle (lights on at 0700 h). All experimental protocols were approved by the Animal Care and Use Committee at the University of Kentucky, and the handling and care of the animals were in accordance with the *National Institutes of Health Guide for the Care and Use of Laboratory Animals*.

### 2.2 Surgery

Rats were OVX and implanted with a telemetry monitor. To this end, rats were anesthetized with isoflurane and given an injection of 5 mg/kg carprofen (sc; Pfizer Animal Health, New York, NY) to minimize pain followed by an injection of 10 mg/kg Baytril (sc; Bayer, Shawnee Mission, KS). Rats were first bilaterally OVX as previously described (Santollo et al., 2013). Next, the telemetry sensor (Steller Telemetry; TSE Systems) was implanted into the abdominal aorta. To accomplish this, the abdominal aorta was exposed, bulldog clamps were placed on the aorta above and below the renal artery branch for up to 5 min, and the tip of the sensor was inserted into the artery and secured with a cellulose patch and Vetbond (Animal Care Products). The telemetry transmitter was then secured to the muscle wall with nonabsorbable sutures, the abdomen was sutured shut, and wound clips were used to close the skin. After surgery, rats received a 5 ml injection (sc) of 0.9% NaCl. Rats received a second injection of carprofen 24 h later. Seven to 10 days later, rats were implanted with a chronic indwelling cannula into the right lateral ventricle as previously described (Santollo et al., 2013). Again, rats were anesthetized with isoflurane and treated with 5 mg/kg carprofen prior to surgery, given a 5 ml injection of 0.9% sterile NaCl at the end of surgery, and treated 24 h later with 5 mg/kg carprofen. Correct cannula placement was determined 3–5 days later by measuring water intake in response to 10 ng AngII (Phoenix Pharmaceuticals). Only rats that drank at least 5 ml in 30 min were included in the study, which included all rats tested.

### 2.3 Testing protocol

**FIGURE 1:**
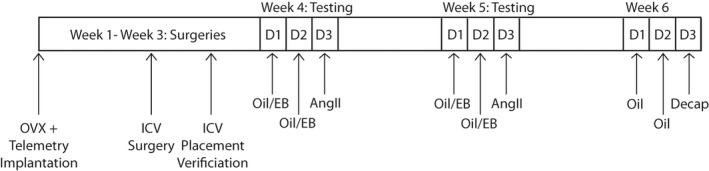
Experimental Timeline. OVX, telemetry implantation, and ICV surgeries occurred during Weeks 1–3. Testing occurred during Weeks 4–5 and brains were collected during Week 6

Behavioral testing began 7–10 days after the second surgery (Figure 1). All behavioral testing occurred in the rats' home cages. Rats were treated with either 0 (oil) or 10 µg estradiol benzoate (EB; Millipore‐Sigma) dissolved in 0.1 ml of peanut oil once a day for 2 days at 1000 h. On day 3 (test day) at 1000 h, food and fluids were removed from the cages and blood pressure, heart rate, and body temperature were monitored for 10 s every 2 min at 200Hz during a 16 min baseline (BL) period. Next, all rats received 1 µl of intracerebroventricular (ICV) injection of 100 ng AngII and then water and 0.26 M NaCl bottles were returned to the cages. Licks, blood pressure, heart rate, and body temperature were recorded for the next 60 min. The experiment was repeated the following week with rats receiving the opposite hormone treatment to achieve a crossover design. The hormone replacement protocol and EB and AngII doses were chosen to be consistent with previous studies, from our group and others, examining the effects of EB on fluid intake in female rats (Jones & Curtis, behavior, 2009,; Kisley et al., 1999; Krause et al., 2003; Santollo et al., 2016). Furthermore, the goal was to examine the inhibitory effect of E2; therefore, we maximized intake by a high dose of AngII. Using the same EB dose across age groups was chosen to be consistent with the field (Lederman et al., 2010; Neal‐Perry et al., 2005, 2008, 2009, 2014).

### 2.4 Intake measures

The distribution of drinking throughout the test was assessed by the use of a contact lickometer that recorded individual licks. The lickometer interfaced with a computer using an integrated USB digital I/O device (National Instruments) and data were processed in Excel. Bottle spouts were behind an electrically isolated metal plate with a 3.175 mm‐wide opening, through which the rat needed to lick to reach the spout, minimizing non‐tongue contacts (i.e., paw) with the spout. Drinking microstructure analysis was processed in Excel. Bursts were defined as at least two licks with an inter‐lick interval of no more than 1 s and burst size was defined as the average number of licks within a burst (Spector et al., 1998).

### 2.5 Tissue collection

The week after behavioral testing was complete, a subset of rats was treated once a day for 2 days with oil (sc). Twenty‐four hours after the second injection, rats were lightly anesthetized with isoflurane, decapitated, their brains were removed, flash‐frozen with 2‐methylbutane (Millipore‐Sigma), and stored at −80°C until further processing. The anteroventral region of the third ventricle (AV3V), subfornical organ (SFO), and paraventricular nucleus of the hypothalamus (PVN) regions of the brain were obtained by slicing 300 µm coronal sections on a cryostat and then taking midline punches (0.5–1.5 mm in diameter) from each brain region as previously described (Santollo et al., 2014). The AV3V and SFO punches (0.5–1 mm) were taken from three serial sections and the PVN punches (1.5 mm) were taken from two serial sections. Tissue punches were stored at −80°C until processing.

### 2.6 cDNA synthesis and Real‐Time‐PCR

Real‐time polymerase chain reaction (PCR) was used to quantify estrogen receptor (ER) and angiotensin type 1 receptor (AT1R) expression in the AV3V, SFO, and PVN as a function of age. DNA‐free total RNA was purified using the E.Z.N.A. MicroElute Total RNA Kit (Omega Bio‐Tek Inc), including a deoxyribonuclease step. Reverse transcription (RT) was performed with 100 ng (SFO and PVN) or 200 ng (AV3V) of RNA using the iScript cDNA Synthesis Kit (Bio‐Rad). Real‐time PCR was carried out using the SYBR GREEN gene master mix (Bio‐Rad) according to the manufacturer's instructions. 18s was used as the housekeeping gene. The primer sequences used were ERα sense, 5′‐TTTCTTTAAGAGAAGCATTCAAGGA, antisense, 5′‐TTATCGATGGTGCATTGGTTT, ERβ sense, 5′‐CTGTGTGGCCATAAAATCAACCT, antisense, 5′‐AGGCAGGAATGCGAAATGAG, GPER sense, 5′‐CCTCAACACTCACACACTCTGG, antisense, 5′‐GATGTCTGGGCTGGTGCT, AT1R sense, 5′‐CGGCCTTCGGATAACATGA, antisense, 5′‐CCTGTCACTCCACCTCAAAACA, 18s sense, 5′‐CACGGGTGACGGGGAATCAG, antisense, and 5′‐CGGGTCGGGAGTGGGTAATTTG, which were chosen based on previous reports (Meitzen et al., 2019; Sample et al., 2012; Santollo et al., 2016; Vaucher et al., 2014). Due to the differential expression patterns of ER subtypes throughout the brain, ERα and GPER were analyzed in the SFO, ERα and ERβ were analyzed in the AV3V, and ERβ and GPER were analyzed in the PVN (Hazell et al., 2009; Shughrue et al., 1997). AT1R expression was analyzed in all brain regions.

### 2.7 Data analysis

Data are presented as means ± SEM throughout. Statistical analyses were performed using Statistica (StatSoft). Total licks, licks for water, and licks for 0.26 M NaCl in 15‐min time intervals (Buyuk et al., 2010) were analyzed with a three‐factor ANOVA (age = between variable and hormone and time = within variables). Licks for water and licks for 0.26 M NaCl across the test period were also analyzed with a two‐factor ANOVA (age = between variable and hormone = within variable). *T*‐tests were used to determine the effect of EB on burst size and burst number within time intervals identified in the lick analysis as being influenced by EB treatment. If no bursts occurred, the burst size could not be calculated, and those subjects were not included in the burst size analysis. This reduced the sample size to 10 middle‐aged rats and nine old rats in the analysis of burst size. Blood pressure was collected and processed with the NOTOCORD‐hem Software package (Newark, NJ). Mean arterial pressure (MAP) and heart rate in 16‐min bins were analyzed by examining the area under the curve (AUC) with a three‐factor ANOVA (age = between variable and hormone and time = within variables). Average body temperature during the 16‐min bins was analyzed with a three‐factor ANOVA (age = between variable and hormone and time = within variables). RT‐PCR values were calculated using the ΔΔCT quantification method with 18s as the normalizing housekeeping gene. ER and AT1R expression as a function of age was analyzed with a one‐way ANOVA for each receptor in each brain region. Data points for SFO ERα and GPER expression were not normally distributed; therefore, the Kruskal–Wallis test was used instead of a one‐way ANOVA. Duncan's multiple range post hoc tests were used throughout to determine group differences after significant main or interaction ANOVA effects and when a priori differences were predicted regarding hormone × age × time effects. Effect sizes were calculated using Cohen's *d* for *t*‐tests (*µ*_1_ − *µ*_2_/SD) and eta squared (*η* ^2^ = SS_effect_/SS_total_) was calculated to determine effect sizes for ANOVA.

## 3 RESULTS

### 3.1 Licking behavior

**FIGURE 2:**
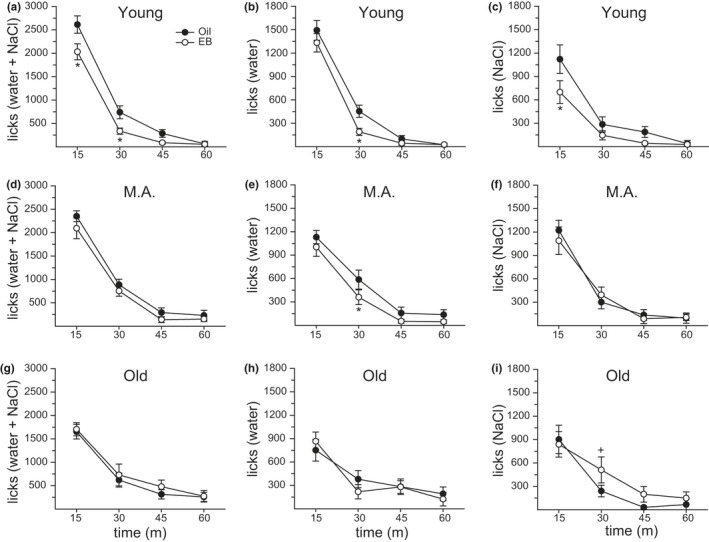
Age influenced the anti‐dipsogenic and anti‐natriorexigenic effects of E2. (a) In young rats, estradiol benzoate (EB) treatment reduced the total number of licks during the first and second 15 min of testing. (b/c) This change was driven by an EB‐mediated reduction in 0.26 M NaCl licks during the first 15 min of testing and a reduction in water licks during the second 15 min of testing. (d) In middle‐aged rats, EB treatment had no effect on the total number of licks, (e) but did reduce licks for water during the second 15‐min of testing. (f) There was no effect of EB treatment on 0.26 M NaCl licks in middle‐aged rats. (g/h) In old rats, EB treatment had no effect on the total number of licks or licks for water. (i) EB treatment enhanced licks for 0.26 M NaCl during the second 15 min of testing. Sample size = 11–12/group. Three‐factor ANOVA: *Less than oil treatment, *p* < 0.05. +Greater than oil treatment, *p* < 0.05

Noncumulative total licks (water +0.26 M NaCl), licks for water, and licks for 0.26 M NaCl were analyzed with age, hormone, and time as variables (Figure 2). Because the effects of central AngII are known to be rapid and transient (Daniels et al., 2009; Santollo et al., 2016), we analyzed these data in 15‐min intervals (bins) to capture both the immediate effects on intake (0–30 min) and any longer lasting or rebound effects on intake (31–60 min). Total licks were influenced by the main effects of hormone (*F*_1,32_ = 3.81, *p* = 0.049, *η* ^2^ = 0.01) and time (*F*_3,96_ = 320.12, *p* < 0.0001, *η* ^2^ = 0.73) and interactions between hormone × age (*F*_2,32_ = 3.08, *p* = 0.049, *η* ^2^ = 0.01) and time × age (*F*_6,96_ = 6.78, *p* = 5.19E^−06^, *η* ^2^ = 0.03). EB reduced licks (*p* = 0.049). Most licks occurred during the first 15 min of the test session (*p* = 0.0001) and licks during the second 15 min of the test session (15–30 min) were greater than during the third (30–45 min) or fourth (45–60 min) 15‐min bins (*p* = 0.0001). EB reduced total licks in young, but not middle‐aged or old rats (*p* = 0.018). In addition, young and middle‐aged rats made more licks during the first 15 min of the test session than old rats (*p* = 0.0002). During the second 15 min of the test session, middle‐aged rats made more licks than young rats (*p* = 0.049). A priori, we predicted that EB would reduce total licks in young, but not aged rats, early in the test period when intake is highest (Santollo et al., 2016). In support of this hypothesis, EB reduced total licks during the 0–15‐ and 15–30‐min periods in young rats (*p* = 0.011; Figure 2a) but had no effect in either middle‐aged (Figure 2d) or old rats (Figure 2g). In summary, licks for water +0.26 M NaCl were reduced by EB during the first 30 min of the test session only in young rats.

Licks for water were influenced by the main effects of hormone (*F*_1,32_ = 14.42, *p* = 0.0006, *η* ^2^ = 0.01) and time (*F*_3,96_ = 147.67, *p* < 0.0001, *η* ^2^ = 0.60) and an interaction between age × time (*F*_6,96_ = 8.56, *p* < 0.0001, *η* ^2^ = 0.07). EB treatment reduced water intake (*p* = 0.0006). Water intake was greatest during the first 15 min of the test period (*p* = 0.0001) and intake during the second 15 min of testing was greater than in the third or fourth 15‐min bins (*p* = 0.0002). During the first 15 min of the test session, water intake was greatest in the young group, with the middle‐aged group consuming more than the old group (*p* = 0.005). During the third 15 min of the test session, intake was greater in old, compared to young, rats (*p* = 0.043). A priori, we predicted that EB would reduce licks for water in young, but not aged rats, early in the test period when intake is highest (Santollo et al., 2016). In partial support of this hypothesis, EB reduced intake in young and middle‐aged rats. This reduction occurred during the 15–30 min period in both young (*p* = 0.027; Figure 2b) and middle‐aged rats (*p* = 0.049; Figure 2e). There was no effect of EB treatment on intake at any time in old rats (*p* = n.s; Figure 2h). Analysis of total water licks during the test session as a function of age × hormone revealed similar findings. Total licks for water were influenced by the main effect of hormone (*F*_1,2_ = 14.42, *p* = 0.0006, *η* ^2^ = 0.11) and post hoc analysis, based on our a priori predictions, demonstrated that in young and middle‐aged, but not old, rats EB treatment reduced licks for water (*p* = 0.0157). In summary, licks for water were reduced by EB during 15–30 min of the test session, but only in young and middle‐aged rats.

Licks for 0.26 M NaCl were influenced by the main effect of time (*F*_3,96_ = 97.62, *p* < 0.0001, *η* ^2^ = 0.50) and interactions between hormone × age (*F*_2,32_ = 3.51, *p* = 0.042, *η* ^2^ = 0.01) and hormone × time (*F*_3,96_ = 3.05, *p* = 0.032, *η* ^2^ = 0.01). Intake of 0.26 M NaCl was greatest during the first 15 min of the test period (*p* = 0.0001) and intake during the second 15 min of testing was greater than in the third or fourth 15‐min bins (*p* = 0.001). EB treatment reduced intake only in young rats (*p* = 0.036) and only during the first 15 min of the testing period (*p* = 0.003). A priori, we predicted that EB treatment would reduce 0.26 M NaCl intake in young, but not aged rats, early in the test period when intake is highest (Santollo et al., 2016). In partial support of our hypothesis, EB treatment reduced intake in young rats during the first 15 min of testing (*p* = 0.002; Figure 2c). There was no effect of EB at any time in middle‐aged rats (*p* = n.s.; Figure 2f). Surprisingly, EB increased intake in old rats during the second 15 min (15–30 min) of the test (*p* = 0.049; Figure 2i). Analysis of total of 0.26 M NaCl licks during the test session did not reveal any main or interactive effects. In summary, licks for 0.26 M NaCl were reduced by EB during the first 15 min of testing in young rats, while EB treatment increased licks for 0.26 M NaCl during the second 15 min of testing in old rats.

### 3.2 Licking microstructure

**FIGURE 3:**
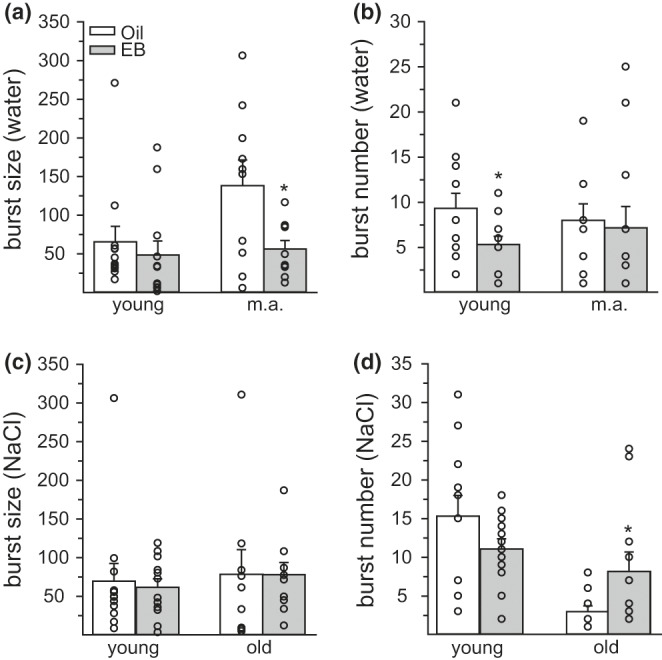
The inhibitory effect of estradiol benzoate (EB) on drinking microstructure was influenced by age. (a/b) EB reduced licks for water in young rats during the second 15 min of testing, by reducing the burst number, but not the burst size. However, EB reduced licks for water in middle‐aged rats by reducing burst size, not burst number. (c/d) In young rats, EB reduced licks for 0.26 M NaCl during the first 15 min of testing; however, there was no significant reduction in either burst size or burst number. In old rats, EB increased licks for 0.26 M NaCl during the second 15 min of testing, which was mediated by an increase in burst number, with no change in burst size. Sample size = 11–12/group. *t*‐test: *Less than oil treatment, *p* < 0.05. ^+^Greater than oil treatment, *p* < 0.05

To determine how EB influenced water and 0.26 M NaCl intake, burst size and burst number were analyzed within the time frames identified above where EB altered the licking behavior. In young and middle‐aged rats, EB reduced licks for water during the second 15 min (15–30 min) of the testing period. At this time, EB had no effect on burst size (*t*_11_ = 0.66, *p* = n.s., *d* = 0.26; Figure 3a), but significantly reduced burst number (*t*_11_ = 2.51, *p* = 0.029, *d* = 0.80; Figure 3b) in young rats. In middle‐aged rats, EB significantly reduced burst size (*t*_9_ = 2.94, *p* = 0.016, *d* = 0.97; Figure 3a), but had no effect on burst number (*t*_11_ = 0.27, *p* = n.s., *d* = 0.12; Figure 3b). In young rats, EB reduced licks for 0.26 M NaCl during the first 15 min of testing. At this time, EB had no effect on burst size (*t*_11_ = 0.34, *p* = n.s., *d* = 0.26; Figure 3C), and while there was a trend for a decrease in burst number, this did not reach statistical significance (*t*_11_ = 1.69, *p* = 0.119, *d* = 0.57; Figure 2d). In old rats, EB increased licks for 0.26 M NaCl during the second 15 min (15–30 min) of testing. At this time, EB had no effect on burst size (*t*_8_ = 0.10, *p* = n.s., *d* = 0.50; Figure 3c), but significantly increased burst number (*t*_10_ = 2.24, *p* = 0.048, *d* = 0.76; Figure 3d). There were no other significant differences in either burst size or burst number for either water or 0.26 M NaCl intake during any other time interval in any age group (*p* = n.s.). In summary, water intake was reduced by a change in burst number in young rats, in contrast, to a change in burst size in middle‐aged rats, while 0.26 M NaCl intake was increased by a change in burst number in old rats.

### 3.3 Physiological Measures: MAP, heart rate, body temperature

**FIGURE 4:**
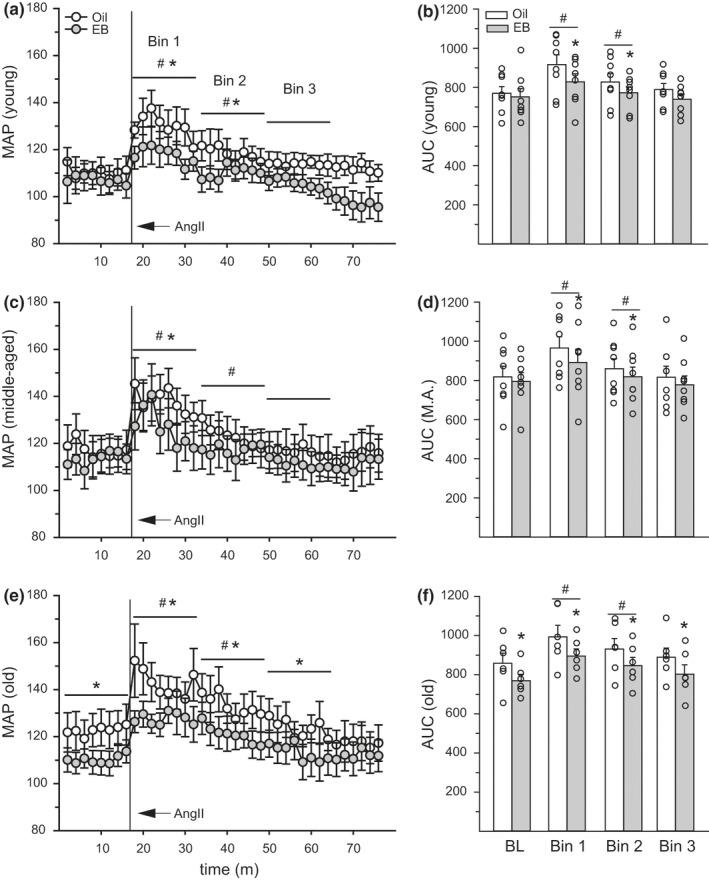
Estradiol benzoate (EB) treatment reduced blood pressure. In (a/b) young, (c/d) middle‐aged, and (e/f) old rats, AngII increased mean arterial pressure (MAP) during the first 32 min of the test period (Bins 1 and 2), compared to BL. MAP was reduced, in EB‐ compared to oil‐treated rats, during Bins 1 and 2 in young rats, Bin 1 in middle‐aged rats, and Bins 1–3 in old rats. In old rats, EB treatment also reduced BL MAP. Sample size = 6–8/group. Three‐factor ANOVA: *Less than oil‐treatment, *p* < 0.05. ^#^Greater than BL, *p* < 0.05

Blood pressure was sampled every 2 min; therefore, data were analyzed in 16‐min bins, instead of 15‐min bins as with the lick data. Our analysis, therefore, included a 16‐min BL bin and three 16‐min bins (Bins 1–3) post‐AngII treatment. MAP from the entire 60‐min test period is graphed (Figure 4), although the final 12 min were excluded from the ANOVA to ensure equal bin sizes for analysis. Importantly, MAP returned to BL levels after ~30 min post‐AngII treatment, providing additional support for excluding the final 12 min of sampling from the analysis. Sample sizes are reduced in the blood pressure analyses (*n* = 6–8/age group) due to technical issues regarding the patency of the telemetry sensors. Analysis of AUC for MAP revealed the main effect of hormone (*F*_1,19_ = 24.638, *p* = 0.00008, *η* ^2^ = 0.05) and time (*F*_3,57_ = 53.484, *p* = 1.11E^−16^, *η* ^2^ = 0.12; Figure 4). As expected, EB treatment reduced MAP (*p* = 0.0002). MAP was increased, compared to BL, 0–16 (Bin 1) and 16–32 (Bin 2) min after ICV infusion of AngII (*p* = 0.0001). A priori, we hypothesized that EB treatment would reduce MAP in young, but not middle‐aged and old rats, immediately after ICV AngII infusion when MAP is the highest (Jonklaas & Buggy, 1984). We found, however, that after ICV AngII infusion, EB treatment reduced MAP during Bin 1 in all three age groups, during Bin 2 in young and old rats, and in Bin 3 in old rats (*p* = 0.001). Furthermore, EB treatment reduced BL MAP in old rats (*p* = 0.002; Figure 4e). Because EB reduced BL MAP in old rats, we next performed an analysis on the delta MAP (change from BL). Delta MAP was only influenced by the main effect of time (*F*_2,38_ = 59.92, *p* = 1.78E^−12^, *η* ^2^ = 0.32). The change in MAP was greatest during Bin 1 and the change in MAP during Bin 2 was greater than during Bin 3. Testing the same a priori predictions as above, we found that EB treatment reduced delta MAP in young and middle‐aged, but not old, rats during the first 16 min after AngII treatment (*p* = 0.03). To summarize, regardless of age, MAP increased after ICV AngII. EB influenced MAP in all age groups, but in young and middle‐aged rats this occurred through a reduction in the pressor response, while in old rats this occurred through a reduction in BL MAP.

Heart rate and body temperature were also recorded and analyzed in 16 min bins (BL, Bins 1–3). Heart rate was influenced by the main effect of age (*F*_2,29_ = 7.14, *p* = 0.004, *η* ^2^ = 0.15) and time (*F*_3,57_ = 28.10, *p* = 2.76E^−11^, *η* ^2^ = 0.20), and interactions between age × time (*F*_6,57_ = 2.99, *p* = 0.013, *η* ^2^ = 0.04) and hormone × time (*F*_3,57_ = 2.91, *p* = 0.043, *η* ^2^ = 0.01; Table 1). Heart rate was higher in young, compared to middle‐aged animals (*p* = 0.01) and higher during BL and Bin 1 than Bins 2 and 3 (*p* = 0.0001). Regardless of hormone, heart rate was higher during BL and Bin 1, compared to Bins 2 and 3 in young and middle‐aged (*p* = 0.004), but not old rats (p = n.s.). Finally, regardless of age, HR was greater in EB‐treated rats during Bin 2. Body temperature was influenced by the main effect of time (*F*_3,57_ = 94.9, *p* < 0.00001, *η* ^2^ = 0.30) and interactions between and age × time (*F_6_*_,57_ = 4.70, *p* = 0.0006, *η* ^2^ = 0.03) and hormone × time (*F*_3,57_ = 5.4, *p* = 0.002, *η* ^2^ = 0.01; Table 1). Body temperature was greatest during the BL period and was significantly reduced with each subsequent time bin (*p* = 0.008). Young rats had higher body temperature during BL and Bin 1 compared to old rats (*p* = 0.023). Finally, EB treatment reduced the body temperature during the BL period (*p* = 0.017). EB‐treated rats, however, had higher body temperature compared to oil‐treated rats, during Bins 2 and 3 (*p* = 0.044).

**TABLE 1:** Heart rate and body temperature

Group	Heart rate (HR)				Body temperature (*T*_b_)			
	Baseline	Bin 1	Bin 2^€^	Bin 3^€^	Baseline	Bin 1*	Bin 2**	Bin 3***
Young‐Oil	405.89 ± 8.15	399.32 ± 6.99	362.87 ± 7.54^+^	356.63 ± 9.35^+^	38.646 ± 0.18÷	38.188 ± 0.202^÷^	37.645 ± 0.206	37.6487 ± 0.209
Young‐EB	392.19 ± 16.56	387.67 ± 15.48	361.92 ± 11.57^# +^	347.44 ± 11.01^+^	38.543 ± 0.16÷^	38.367 ± 0.24^÷^	37.367 ± 0.24'	37.589 ± 0.17'
MA‐Oil^δ^	369.85 ± 9.81	363.30 ± 7.17	332.19 ± 5.00^+^	331.19 ± 4.38^+^	38.519 ± 0.210	38.056 ± 0.22	37.540 ± 0.273	37.236 ± 0.23
MA‐EB^δ^	367.36 ± 2.69	363.64 ± 6.17	345.95 ± 5.12^# +^	327.25 ± 5.11^+^	38.372 ± 0.16^^^	38.095 ± 0.11	37.668 ± 0.12'	37.380 ± 0.14'
Old‐Oil	360.28 ± 9.87	367.04 ± 5.77	343.19 ± 8.29	348.49 ± 9.26	38.113 ± 0.20	37.728 ± 0.16	37.360 ± 0.18	37.334 ± 0.21
Old‐EB	357.34 ± 5.83	370.44 ± 4.78	364.39 ± 12.03^#^	353.13 ± 11.98	37.839 ± 0.16^^^	37.632 ± 0.22	37.541 ± 0.26'	37.629 ± 0.16'

Note

HR: Age: ^δ^Young > middle‐aged; HR: Time: ^€^BL + Bin 1 > Bins 2 and 3; HR: Time × Age: ^+^Baseline and Bin 1 > Bin 2 and 3; HR: Hormone × Time: #EB > Oil, *p* < 0.05.

*T*_b_: Time: *Less than BL, **Less than BL and B1, ***Less than BL, B1, B2; *T*_b_: Age × Time ^÷^Young > Old; *T*_b_: Hormone × Time: ^Oil > EB; 'Oil < EB, *p* < 0.05.

### 3.4 Gene expression

**FIGURE 5:**
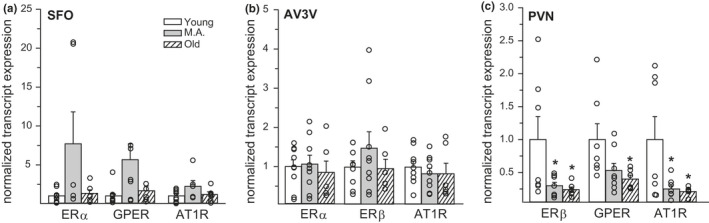
Age influenced estrogen receptor (ER) and angiotensin type 1 receptor (AT1R) expression in discrete brain regions. (a) In the subfornical organ (SFO), there was no influence of age on ERα, GPER, or AT1R expression. (b) In the AV3V, there was no influence of age on ERα, ERβ, or AT1R expression. (c). In the paraventricular nucleus of the hypothalamus (PVN), both middle‐aged and old rats had significantly less ERβ and AT1R expression, compared to young rats. Old rats also had significantly less GPER expression than young rats. Sample size = 6–9/group. Kruskal–Wallis Test (SFO: ERα and GPER)/one‐way ANOVA: *Less than young, *p* < 0.05

The expression of ERα, ERβ, GPER, and AT1R was quantified as a function of age in the SFO, AV3V, and PVN. Again, due to the differential expression patterns of ER subtypes throughout the brain (Hazell et al., 2009; Shughrue et al., 1997), ER analysis was limited to the specific subtypes expressed in each brain region. Age had no effect on ERα (*H*_2,21_ = 2.04, *p* = n.s., *η* ^2^ = 0.26), GPER (*H*_2,21_ = 3.21, *p* = n.s., *η* ^2^ = 0.30), or AT1R (*F*_2,18_ = 2.02, *p* = n.s., *η* ^2^ = 0.18) expression in the SFO (Figure 5a). Age had no effect on ERα (*F*_2,21_ = 0.20, *p* = n.s., *η* ^2^ = 0.02), ERβ (*F*_2,21_ = 0.87, *p* = n.s., *η* ^2^ = 0.08), or AT1R (*F*_2,21_ = 0.31, *p* = n.s., *η* ^2^ = 0.03) expression in the AV3V (Figure 5b). Age influenced ERβ (*F*_2,18_ = 4.19, *p* = 0.032, *η* ^2^ = 0.32), GPER (*F*_2,18_ = 3.68, *p* = 0.046, *η* ^2^ = 0.29), and AT1R (*F*_2,18_ = 4.71, *p* = 0.023, *η* ^2^ = 0.34) expression in the PVN (Figure 5c). The expression of ERβ and AT1R was reduced in middle‐aged and old rats, compared to young rats (*p* = 0.028 and 0.018, respectively). The expression of GPER was reduced in old rats, compared to the young rats (*p* = 0.022), and just failed to reach a statistically significant reduction in middle‐aged rats (*p* = 0.057). Three tissue samples were not included in the SFO and PVN analysis due to issues related to tissue collection or mRNA extraction.

## 4 DISCUSSION

Although the anti‐dipsogenic and anti‐natriorexigenic effects of E2 have been well characterized (Santollo & Daniels, 2015b), to our knowledge, this is the first report to directly test the effect of aging on the fluid intake effects of E2. Our results demonstrate that while middle‐aged rats retain the anti‐dipsogenic effect of E2, it is lost in old rats. In addition, the anti‐natriorexigenic effect of E2 that was observed in young rats was lost in both middle‐aged and old rats. E2 also dampens the rise in blood pressure in response to the central treatment of AngII (Jonklaas & Buggy, 1984). Here, we demonstrate that E2 modulates blood pressure in young, middle‐age, and old rats. Finally, we observed a reduction in ER and AT1R expression within the PVN as a function of age. Together these results demonstrate that aging impedes E2's fluid intake effects which correlate with a reduced capacity for ER and AT1R signaling in the PVN.

While the anti‐dipsogenic effect of E2 was only lost in old rats, the anti‐natriorexigenic effect was lost by middle age. This suggests that disturbances in the fluid intake effects of E2 begin in middle age but are most profound in old rats. This finding also highlights an important difference in the control of fluid intake by E2 and provides further evidence that E2 has separable effects on the controls of water and saline intake (Santollo et al., 2016), instead of a more general reduction in overall consumption. This will help guide our investigation of the mechanisms by which E2 acts to reduce water and saline intake. This finding also adds to the literature demonstrating age‐related changes in E2 effects (Buyuk et al., 2010; Santollo et al., 2012; Wise et al., 1991). Our findings here highlight the necessity for additional research into whether other effects of E2 on homeostasis, such as locomotor activity and metabolism, are altered by aging.

Surprisingly, the drinking microstructure underlying the anti‐dipsogenic effect of E2 changed as a function of age. In young rats, EB treatment reduced water intake through a reduction in burst number. In middle‐aged rats, EB treatment, in contrast, reduced water intake through a reduction in burst size. Based on decades of work analyzing licking microstructure that associates changes in burst number with postingestive feedback and changes in burst size with orosensory feedback (Davis, 1989; Davis et al., 1999), the present data suggest that EB alters postingestive signals in young rats and orosensory signals in middle‐aged rats. How aging influences the underlying drinking microstructure by which E2 reduces water intake is unclear and will require additional research. A first step, however, will be to identify how E2 alters postingestive signals in young rats before we can understand how aging alters E2‐mediated changes in drinking microstructure. This shift, however, may help elucidate how the anti‐dipsogenic effect is ultimately lost, as observed here in the old rats. There was no significant change in the drinking microstructure for 0.26 M NaCl intake in young rats after EB treatment, although we did observe a trend (*p* = 0.12) for a reduction in burst number. Finally, the unexpected enhancement of 0.26 M NaCl intake after EB treatment in old rats was mediated by an increase in the burst number. This suggests that changes in postingestive feedback may enhance 0.26 M NaCl intake after EB treatment in aged animals.

Independent of E2 effects, we found that aging reduced fluid intake in females, which supports research from other groups. Previous work has demonstrated that intake stimulated by water deprivation, isoproterenol, thermal dehydration, hypertonic saline, and furosemide is reduced in aged female rats (25–40 months old) compared to young (4–5 months) or middle‐aged (9–10 months) female rats (Begg et al., 2012; Hardy et al., 2018; Martin et al., 1985). Begg et al., in contrast, did not find an effect of aging on intake stimulated by peripheral AngII (Begg et al., 2012), yet our data demonstrate an age‐related reduction in AngII‐stimulated intake. The discrepancy here could be due to differences in route of AngII administration, central versus peripheral, or differences in the ages of the old groups, 18 months versus 30 months. Regardless, Begg et al. did observe a diminished drinking response after 24‐h water deprivation and furosemide treatment (Begg et al., 2012), both of which stimulate drinking in part due to endogenous AngII (Daniels & Fluharty, 2009). Our study provides further evidence that aging diminishes drinking responses in female rats. It is tempting to speculate that the reduced dipsogenic effect of AngII in older animals contributed to the lost fluid intake effects of E2 that were observed here. Future studies will be necessary to test this idea.

In addition to its dipsogenic effect, central AngII increases blood pressure through increased sympathetic output and vasopressin release, an effect dampened by E2 (Jonklaas & Buggy, 1984). Unlike the fluid intake effects of E2, we found that E2 reduced blood pressure in all three age groups. There were, however, differences in this effect, whereby E2 reduced the pressor response that occurred after the ICV AngII injection in young and middle‐aged rats, but reduced BL blood pressure in old rats. This supports previous research demonstrating that E2 reduces systolic blood pressure in middle‐aged (9 months) OVX rats (Clark et al., 2004) and reduces MAP in middle‐aged (10–12 months) OVX Dahl salt‐sensitive rats (Hinojosa‐Laborde et al., 2004). Our results, however, are only in partial agreement with another study, which also demonstrated a similar pressor response in intact and OVX aged mice. In this study, however, no differences in BL MAP were detected, unlike in our study (Mirabito et al., 2014). While this suggests that the ability of E2 to influence blood pressure is lost in aged mice, E2 was not experimentally manipulated or measured. It is, therefore, possible that hormone levels may have been similar in the aged (16 months) intact and OVX mice. In addition, species‐related difference could contribute to the discrepancies. How EB reduces the pressor response caused by central AngII treatment is unclear. Future studies, therefore, should examine how E2 may augment pressor‐related changes induced by vasopressin and sympathetic activation. Finally, of note drinking increases blood pressure in males, but how this effect may be influenced by aging in females is unknown (Hoffman et al., 1977). Follow‐up studies should examine the pressor response to AngII in rats without access to fluids to disentangle the effects of age, E2, and drinking on the pressor response.

Handling stress related to the ICV injection could have increased blood pressure, but because it is well established that ICV AngII increases MAP (Jonklaas & Buggy, 1984; Vento et al., 2012), we did not include a vehicle injection. Therefore, caution should be taken when interpreting our data as age could augment any effect the injection itself had on the pressor response observed after AngII treatment. This concern, however, is mitigated by the fact that stress is associated with an increase in heart rate and body temperature (Kirby et al., 1989; Long et al., 1990), neither of which we observed. Heart rate was initially unchanged post‐AngII infusion and, as reported by others, decreased later in the testing period, likely the result of the baroreflex (Casto & Phillips, 1984). Furthermore, we found a reduction in body temperature after ICV treatment, replicating the previously reported hypothermic effect of AngII (Huang et al., 1985; Lin et al., 1980). Furthermore, while we replicated the previous finding that EB treatment reduces BL body temperature (Dacks & Rance, 2010; Williams et al., 2010), we report the novel finding that EB attenuated the hypothermic effect of AngII. This supports the more general hypothesis the E2 action defends metabolic homeostasis.

The lamina terminalis and key areas of the hypothalamus, such as the PVN, play critical roles in the regulation of fluid balance. Indeed, both AT1R and ER are expressed in these areas of the brain (Allen et al., 2000; Hazell et al., 2009; Shughrue et al., 1997), where E2 action influences fluid balance (Curtis, 2009; Santollo & Daniels, 2015b). Because we observed a loss of the fluid intake effects of E2 as a function of age, we asked whether there are changes in ER and AT1R expression in fluid relevant brain regions in older animals. Indeed, in the PVN, we observed a decrease in ERβ and GPER, a brain area critically involved in the drinking effects of E2 (Tanaka et al., 2002). We also observed a decrease in AT1R expression in the PVN as a function of age.

Future studies are needed to understand the functional significance of the reduction in ER and AT1R expression in the PVN. We have previously demonstrated that both ERβ and GPER signaling underlie the anti‐natriorexigenic effect of E2 (Santollo & Daniels, 2015a; Santollo et al., 2016). Future studies, therefore, should examine how the reduced expression of these receptors in the PVN may contribute to the loss of the anti‐natriorexigenic effect of E2 in middle‐aged and old rats. While a reduction in ER expression is one possible mechanism for the lost fluid‐intake effects of E2, many other age‐related changes could contribute as well. For example, the ability of EB to penetrate neuronal tissue as a function of age should be considered. In addition, a limitation of our study was that it utilized single doses of EB and AngII. A thorough examination of the dose–response curves as a function of age may reveal additional insights into the underlying mechanisms by which the fluid intake effects of E2 are augmented in aging rats. Since AT1R expression changed as a function of age, a related question is whether brain‐derived AngII levels are influenced by aging. Future studies should also examine whether components of AngII synthesis, such as angiotensinogen, renin, and ACE, have altered neuronal expression as a function of age.

To our knowledge, this is the first report to examine age‐related changes in ERα and ERβ expression with the lamina terminalis and age‐related changes in the expression of GPER in any nuclei. Two studies, however, have reported no age‐related change in ERβ expression within the PVN (Wilson et al., 2002; Yamaguchi‐Shima & Yuri, 2007). The discrepancy between these studies and ours is unclear but could be due to methodological differences as rats were OVX here but were exposed to E2 in the other reports. Future studies comparing ER expression in oil and EB‐treated rats as a function of age will be an important next step. Future studies should also examine how ratios of the different ER proteins may change as a function of age within these different brain regions.

This is the first report to demonstrate that the fluid intake effects of E2 are lost as a function of age in OVX rats. This finding is in juxtaposition to E2's ability to reduce blood pressure in young, middle‐aged, and old rats, although age‐related changes in how E2 reduced MAP were observed. These findings highlight areas for future research to understand underlying mechanisms regarding how aging influences behavioral responses to E2. Specifically, we propose that studies on the functional significance of reduced ER and AT1R expression in the PVN is a logical starting point for investigation given the data presented here. Fluid intake and the defense of body fluid homeostasis, however, are complex biological processes. Therefore, it is likely that multiple age‐related changes influence the fluid intake effects of E2. These findings further highlight the need for more research in understanding how body fluid homeostasis is defended in women across the lifespan.

## CONFLICT OF INTEREST

The authors have no conflicts of interest, financial or otherwise, to disclose.

## AUTHOR CONTRIBUTION

Jessica Santollo designed and helped perform the experiments, analyzed data, and wrote the manuscript. Jason A. Collett and Andrea A. Edwards carried out the experiments and edited the manuscript.

## Data Availability

The data that support the findings of this study are available from the corresponding author upon reasonable request.
